# Negative differential resistance and characteristic nonlinear electromagnetic response of a Topological Insulator

**DOI:** 10.1038/srep18008

**Published:** 2015-12-11

**Authors:** Ching Hua Lee, Xiao Zhang, Bochen Guan

**Affiliations:** 1 Department of Physics, Sun-Yat-Sen University, Guangzhou, China; 2 Institute of High Performance Computing, 1 Fusionopolis Way, Singapore 138632

## Abstract

Materials exhibiting negative differential resistance have important applications in technologies involving microwave generation, which range from motion sensing to radio astronomy. Despite their usefulness, there has been few physical mechanisms giving rise to materials with such properties, i.e. GaAs employed in the Gunn diode. In this work, we show that negative differential resistance also generically arise in Dirac ring systems, an example of which has been experimentally observed in the surface states of Topological Insulators. This novel realization of negative differential resistance is based on a completely different physical mechanism from that of the Gunn effect, relying on the characteristic non-monotonicity of the response curve that remains robust in the presence of nonzero temperature, chemical potential, mass gap and impurity scattering. As such, it opens up new possibilities for engineering applications, such as frequency upconversion devices which are highly sought for terahertz signal generation. Our results may be tested with thin films of Bi_2_Se_3_ Topological Insulators, and are expected to hold qualitatively even in the absence of a strictly linear Dirac dispersion, as will be the case in more generic samples of Bi_2_Se_3_ and other materials with topologically nontrivial Fermi sea regions.

Topological Insulators (TIs) are a new class of materials with a fully insulating gap in the bulk but gapless (conducting) Dirac fermion states on the surface^1^,^2^,^3^, and have garnered tremendous interest in condensed-matter physics, material science and electrical engineering communities^1^,^2^,^3^,^4^,^5^,^6^,^7^,^8^,^9^,^10^,^11^,^12^,^13^,^14^. Recent experimental realizations of TI states in compounds like HgTe, 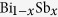 and Bi_2_Te_3_ fueled further enthusiasm in their possible application in devices. The Dirac cones on the surface of 3-dimensional TIs are reminiscent of the Dirac cones in 2-dimensional Graphene, another exotic material which has attracted considerable attention^15^,^16^,^17^,^18^,^19^,^20^,^21^. Notably, Graphene has been theoretically predicted^19^,^20^ and subsequently experimentally shown^21^ to exhibit strong nonlinear electromagnetic response owing to its unique linear Dirac dispersion^15^,^16^,^17^,^18^,^19^,^20^,^21^.

Inspired by these properties of Graphene, we ask if similar, if not more desirable, nonlinear behavior is also present in the TIs. As we will show in this work, the answer is in the affirmative: In fact, the response curve of TIs is even more nonlinear, with an exotic regime of negative differential resistance that persists even in the absence of a strictly linear dispersion. More precisely, the response curve takes the form of an ‘N’ shape with negative differential resistance in the middle segment, similar to the shape of the response curve leading to the Gunn effect in GaAs^22^, but due to a completely different physical mechanism. This suggests an array of potential optoelectronics applications beyond those of Graphene, the Gunn diode and a nonlinear device known as the Bilayer Pseudospin FET (BiSFET)^23^,^24^,^25^.

The enhanced nonlinearity of the response of TI surface states can be understood as follows. A TI heterostructure has two conducting surfaces, the top and bottom surfaces, while a Graphene sheet only has one. Due to the existence of the substrate in the TI heterostructure, structural inversion symmetry has to be broken, leading to a breaking of the degeneracy of the two TI surface states which opens up a Rashba-type spin splitting^26^. This results in an unique Dirac ring bandstructure which exhibits a much stronger nonlinear electromagnetic response than a Dirac cone alone, thereby opening up a venue for interesting physics as well as potential applications.

In this work, we model a TI heterostructure as a Dirac ring system, and analytically and numerically study its semiclassical nonlinear response. We first consider the case of an ideal Dirac ring, i.e. at zero mass gap, temperature and impurities. Next we consider deviations from these ideal conditions, and crucially show that the characteristic features of the response curve remain robust. We further discuss how this semiclassical analysis can be generalized to a more general setting with scattering and/or Rashba-like dispersion, and its implications for the output signal. Finally, we discuss some experimental proposals and engineering applications.

## Results

We first introduce some basic theory on the semi-classical electromagnetic response of a generic Hamiltonian. With that, we present our main results on the characteristic nonlinear response curve of Dirac ring systems. Such systems have been detected in the surface states of thin films of Bi_2_Se_3_ TIs via ARPES experiments^26^, and we will return to discussing the experimental signatures of our results after developing its theory.

### Theory of semiclassical response

Consider a generic system described by a Hamiltonian 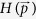 under the influence of a driving field 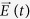. At the semi-classical level, the field shifts the crystal momenta 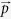 of the partially occupied bands, leading to an induced current


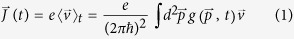


where 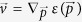 is the canonical velocity and 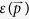 is the eigenenergy for a particular band. In other words, 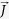 is the expectation value of the current 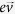 over states weighted by the time-dependent occupation function 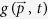. As will be shown rigorously in Sect. 6, the time-dependent occupation function takes the form of the Fermi-Dirac distribution 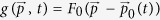 , but with momentum shifted by an *effective* driving impulse 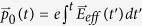. We shall elaborate on exactly how 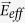 depends on 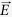 and the scattering time *τ* in Sect. 6. For now, we shall proceed by treating 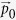 as an external influence, and note that 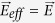 in the limit of zero scattering. This approach considers only intra-band transport processes, and is valid when 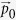 originates from an oscillatory electric field with 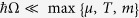, i.e. with frequencies under 20 THz in typical applications. Henceforth, we shall work in units where 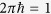 and *k*_*B*_ = 1 for notational simplicity.

If the bands of the hamiltonian are isotropic with eigenenergies 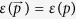 where 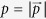, *F*_0_ depends only on *p* via *ε*(*p*) and we can further express 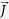 as


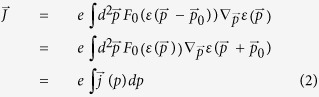


where


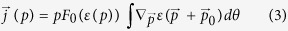


is the contribution from the *p*-momentum shell. This decomposition is particularly useful since 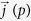 can often be expressed in closed-form, although 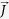 usually cannot be.

**Figure 1:**
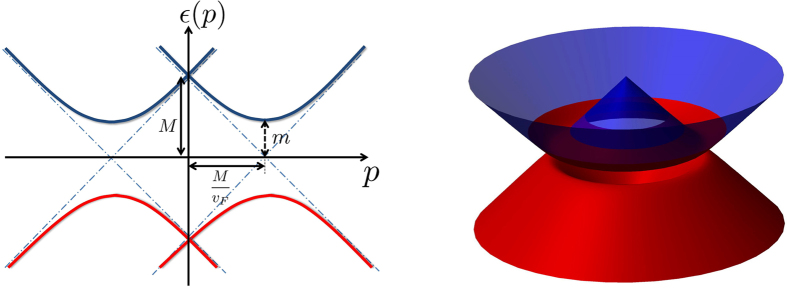
Left) The bandstructure of a Dirac ring system ±*ε*(*p*) given by Eq. 4, with asymptotic canonical velocity (slope) given by 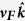. Right) 3D view of the same band structure, with the axis of rotation the energy axis and the plane of rotation the *p*_*x*_, *p*_*y*_ plane. The occupied (red) band is separated from the partially occupied (blue) band by a mass gap 2*m*. They can be obtained by gapping out two intersecting Dirac cone with energy difference of 2*M*. A ring of filled states (red) at chemical potential *μ* > *m* lies in the ring of minima on the valence (blue) band.

We now specialize to the Hamiltonians with a Dirac ring, which is the focus of this work. The energy dispersion of a Dirac ring system takes the form (Fig. 1)


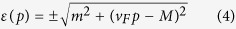


with *v*_*F*_ the Fermi velocity, *m* the (half) gap and 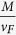 the ring radius. It is known as a Dirac ring system because it is rotationally invariant about 
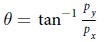
, and has a ring of band minima with linear dispersion at radius 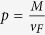. Note that it reduces to the Dirac cone in Graphene when *M* = 0, which was systematically studied in ref. 19. In TI heterostructure realization reported and analyzed in refs 26, 27, 28, 29, *m* is the mass gap induced by interlayer coupling and *M* measures the extent of inversion symmetry breaking.

#### Nonlinear response of Ideal Dirac rings

We shall first study the proptotypical case of *ideal* Dirac rings, where the gap *m* and temperature *T* are both zero. In the limit of small chemical potential 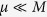, which can be tuned by varying the gate voltage^29^, the filled states on the valence band form a very thin ring bounded by inner and outer Fermi momenta 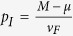 and 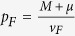, i.e. with radial thickness 2*μ*/*v*_*F*_. Being such a thin ring, its total current is thus well-approximated by 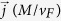 in Eq. 3, which is simple enough to visualize schematically and exactly evaluate analytically (Eq. 15).

The canonical velocity is simply given by


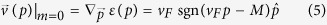


which is a vector of *constant* magnitude *v*_*F*_. It is always pointing in the radial direction, and is positive(negative) outside(inside) the Dirac ring. Such a scenario occurs when the filled states forms a non simply-connected ‘ring-like’ shape instead of a ‘blob-like’ shape. In this case, the lower dimensionality of the ring may allow for some novel kind of ‘destructive interference’ to occur between the contributions on both sides of the ring, and hence lead to negative differential resistance. Before proceeding with the calculations, let us attempt to understand that more intuitively.

**Figure 2:**
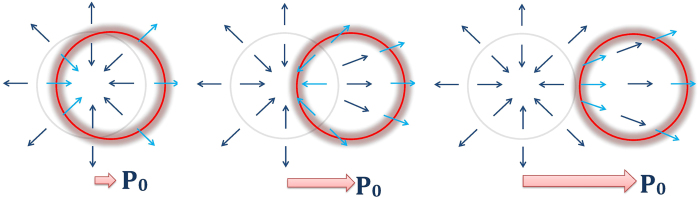
Contributions to the nonlinear response due to 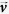 in momentum space. 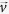
 at filled momentum states contribute to the response, and are colored light blue. For reference, those that do not lie at occupied momentum states are colored dark blue. Left) For small driving impulse *p*_0_, the current 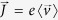 is relatively large as it is contributed by 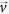 whose horizontal components add constructively (light blue). Middle) As *p*_0_ increases to the range 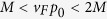, the horizontal components of 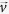 experience a partial cancellation (light blue), thereby lowering the resultant 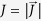. Right) Beyond 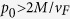, the ring of filled states disentangles with the ring of Dirac nodes (grey). The contributions from 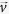 (light blue) add even more strongly, resulting in an even greater *J*. Very importantly, the behavior described here still holds for a ring thickened by (not too large) chemical potential or temperature, and even remain qualitatively true in the presence nonuniformities in 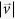, i.e. from nonzero mass.

Without a driving field, 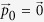 and the ring of filled states lie exactly above the ring of Dirac nodes. On either side of it, the vector field 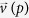 points in equal and opposite directions, thereby resulting in a zero net current in Eq. 33. and 3. Upon a small impulse 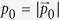 from the driving field, the ring of filled states will be slightly displaced in reciprocal (momentum) space, leading to an imbalance between the contributions of 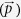 from inside and outside the ring. As illustrated in Fig. 2, 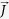 is relatively large for small *p*_0_ because its arises from 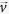 contributions that point in the *same* horizontal direction. As 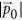 increases to more than half the radius of the ring, contributions from inside the ring *oppose* those from outside the ring, thereby leading to a *decrease* in 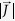. Finally, for *p*_0_ larger than the radius of the ring, the filled states disentangle from the Dirac ring completely, and the 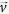 contributions point in *same* horizontal direction again, adding up more strongly than before. As *p*_0_ continue to increase, 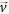 will eventually become parallel, leading to a maximal current 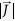 proportional to the size of the ring.

**Figure 3:**
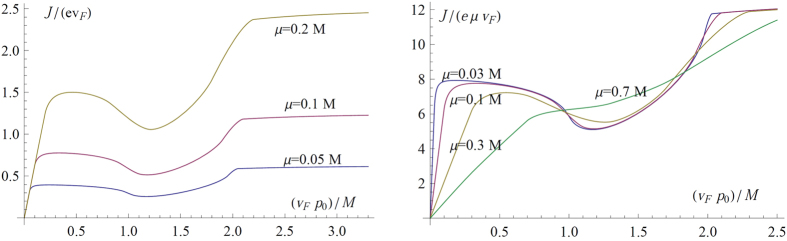
Plots of the current in Eq. 6 against *v*_*F*_*p*_0_/*M* (defined as *Q*^−1^ in Eq. 16), where *p*_0_ is magnitude of the effective driving impulse. *p*_0_ = *eE*_0_*τ* in the DC scattering limit, where *τ* is the scattering time. Left) Behavior of *J*/(*ev*_*F*_) for *μ* = 0.05, 0.1 and 0.2 in units of *M*. We see a strong linear regime at very small *p*_0_, and a region of negative differential resistance 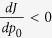 at larger values of *p*_0_ due to destructively interfering 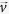. Right) The same plot normalized by *μ*: *J*/(*μev*_*F*_) for *μ* = 0.03, 0.1, 0.3 and 0.7 in units of *M*. We now clearly see the asymptotic properties of the varies curves. As *μ* decreases, the response curve becomes ‘sharper’ due to the decreasingly thickness of the ring of filled states, and eventually converges nonuniformly to *G*(*Q, Q*) (from Eq. 16) as 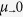.

The above arguments suggest a response curve 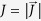 that rises sharply to a moderately large value when *p*_0_ is very small, decreases when 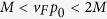, and increases to an even larger value for larger *p*_0_. We identify the decreasing region as the region of negative differential resistance 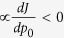. This agrees exactly with analytic expression derived in section and plotted in Fig. 3:


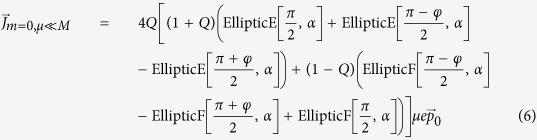


with 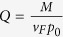, 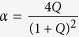, 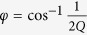, 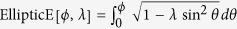 and 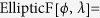 
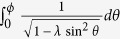
. Note that only the dimensionless combination 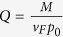 enters the expression.

#### Distortion of a sinusoidal signal

**Figure 4:**
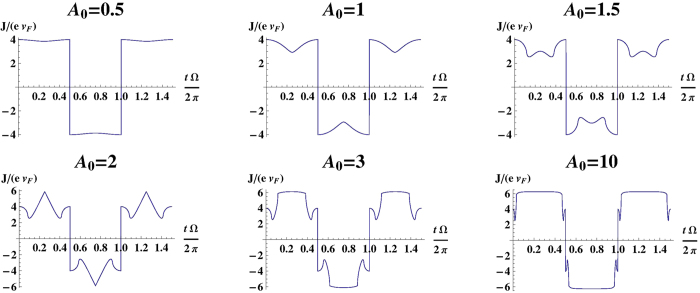
Output currents corresponding to input signals 
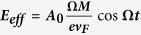
, where *A*_0_ = 0.5, 1, 1.5, 2, 3, 10 for very small *μ*. Note the almost square-wave-like waveform for small and large *A*_0_, where the response curve is mostly flat (Fig. 3). The negative differential resistance part of the response curve produces the interesting lobes in the output current. They will be smoothed out at larger *μ*, nonzero gap or nonzero temperature. In the 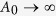 limit, the Dirac ring becomes irrelevant and the response curve reduce to that of Graphene (∝ sgn*p*_0_) in the same limit.

Below, we show how an ideal Dirac ring system distorts periodic signals of different amplitudes. Due to the segment of negative differential resistance in the response curves in Fig. 3, additional kinks and lobes are introduced in the output signal 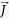. These lead to larger high-frequency components than what can be obtained with Graphene^19^, whose distorted output signal is a square-wave which also appears in the 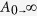 limit of the Dirac ring (Fig. 4).

The extent of the lobes in the output can be quantified by the Fourier coefficients of the output current


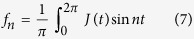


Assuming an input impulse ∝sin *t*, we find that only odd coefficients *f*_2*n*−1_ are nonzero. The distribution of 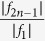 characterizes the frequency multiplication efficacy, which is an important concern in the production of low-power electromagnetic radiation from lower frequencies. This is of particular exigence in the frequency window of 0.3 to 20 THz (commonly known as the Terahertz gap) where, despite a multitude of applications across engineering, material science and medical disciplines^19^,^30^,^31^,^32^,^33^, inexpensive and compact sources for the THz radiation are still lacking.

**Figure 5:**
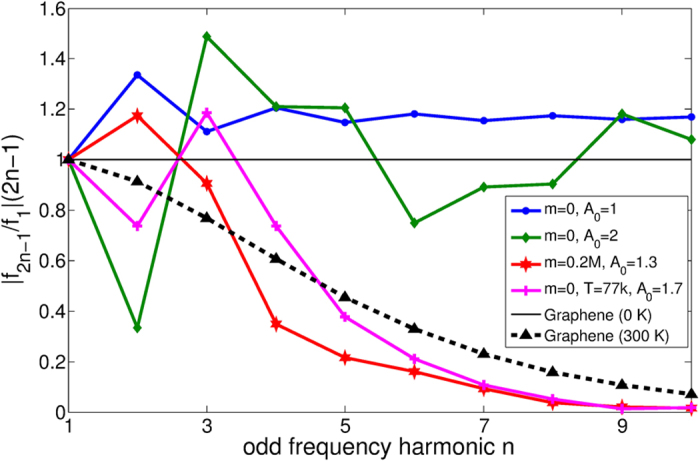
Ratios of the frequency multiplication factor 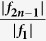 of the Dirac ring with that of zero-temperature Graphene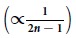. The amplitudes *A*_0_ are given by 
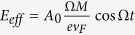
, which give maximal effective impulses 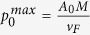. For reference, the continuous black line corresponds to Graphene at zero temperature *T*, which has the maximum possible frequency multiplication factor without negative differential resistance. It is exceeded by that of the gapless, zero-temperature Dirac ring at several different harmonics. This is expected from the appearance of additional, higher-frequency lobes in Fig. 4, where the sharper lobes in the *A*_0_ = 2 curve now manifest themselves as a suppression of the first higher harmonic *f*_3_ relative to the *A*_0_ = 1 case. At nonzero *T* or *m*, the higher harmonics are exponentially suppressed. However, the *n* = 2 or *n* = 3 multiplication factor can still exceed the maximal possible without negative differential resistance (black line), as well as that of Graphene at *T* = 300 *K, μ* = 0 under the same input signal as given in the discussion section.

It is interesting to compare the decay of 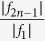 for the Dirac ring with the slowest decay spectrum possible *without* negative differential resistance 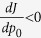. The latter is given by a response curve proportional to sgn *p*_0_, which can be realized^19^ in Graphene at low (or zero) chemical potential and temperature relative to *v*_*F*_*p*_0_. Simple computation yields a harmonic decay profile 
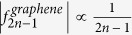
, 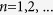. This is compared with that of the Dirac ring for various values of mass gap, temperature and input signal amplitudes in Fig. 5. We see a larger frequency multiplication factor, i.e. larger 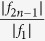 from the gapless Dirac ring across most of the harmonics 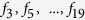. When an energy scale is introduced by either nonzero gap or temperature, *f*_2*n*−1_ decays exponentially. Even then, the frequency multiplication factor still outperforms that of Graphene for the the first few harmonics.

### Response of imperfect Dirac ring materials

So far, we have considered Dirac ring systems with vanishing mass gap *m*, such as those realized in Bi_2_Se_3_ TI thin films heterostructures thicker than 6 quintuple layers (QLs)^26^. To demonstrate the robustness of the regime of negative differential resistance, we shall now analyze Dirac ring systems which possess a gap and correspondingly a departure from perfect linear dispersion. They arise in sufficiently thin TI films where hybridization of the surface states on either side of the TI opens up a gap due to wavefunction overlap^27^,^28^. This had been predicted^27^,^28^ to occur and was indeed observed^26^ in Bi_2_Se_3_ heterostructures thinner than 6 QLs.

Real materials under experimental conditions furthermore experience non-negligible temperature effects and impurity scattering, both of which can undermine the preceding Dirac ring interference analysis. But very importantly, we shall show that the qualitative features of the response curve, particularly the region of negative differential resistance, remain robust. As long as the occupied states still occupy a ring-like region in reciprocal space, we indeed observe:

1. A rapidly increasing response *J* for small *p*_0_;
2. A region of decreasing *J* 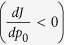 at moderate 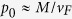, the radius of the ring;
3. An increasing, even larger *J* for larger *p*_0_.

To understand why, it is useful to examine Fig. 2 again. The non-monotonicity of the response (Center) is a consequence of the destructive interference of the contributions of 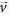 from inside and outside the ring. This is a *generic* feature for a ring of energy minima, since the 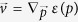, which is always of opposite sides of the ring. In particular, note that it does not depend on the dispersion being linear, although the destructive interference will be less pronounced when the ring is thickened by large *μ* or *m*, or fuzzied by nonzero temperature *T*.

Below, we shall substantiate the above schematic arguments with detailed analyses and numerical results.

#### Effect of nonzero band gap *m*

**Figure 6:**
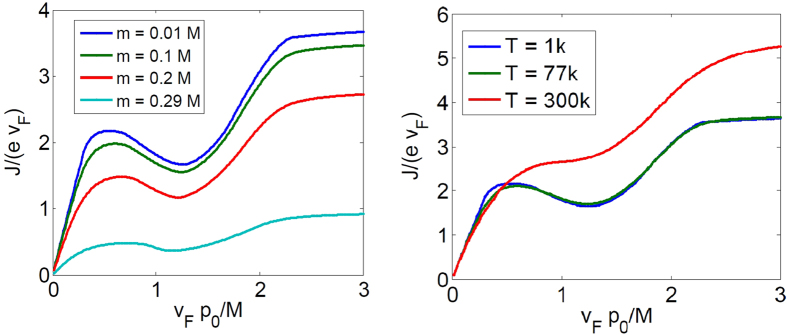
Left) The current response for different values of masses *m*, all at chemical potential *μ* = 0.3 *M* and temperature *T* = 0. As expected, we observe the same qualitative, though ‘rounder’, response curve with increasing *m*. The current diminishes to zero as 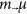, when both 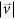 and the thickness of the filled ring diminishes. Right) The current response for different temperatures, with *M* set to 0.1 *eV* and *μ* = 0.3 *M*. There is essentially no difference in the curves at *T* = 1 *K* and *T* = 77 *K* (liquid nitrogen temperature).

In a gapped Dirac ring system, Eq. 4 gives the canonical velocity in a gapped Dirac ring system as


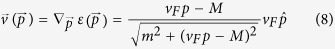


which vanishes linearly near the Dirac ring. As such, we expect a more ‘rounded’ response curve, as shown in Fig. 6 (Left). Note that Eq. 8 also describes the small *p*_0_ response in a Rashba system. The latter, however, has a asymptotically quadratic dispersion which leads to a (rather uninteresting) linear response for large 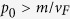, which also suppresses the region of negative differential resistance.

The results for nonzero mass gap *m* are shown in Fig. 6 (Left). The response curves exhibit the same qualitative shape, but with a broader linear response regime for small 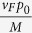, which is studied in more detail in Sect.. Examples of such cases include the bulk states of HgTe^34^ and GaAs^35^ quantum wells with inversion symmetry breaking.

#### Effects of nonzero temperature *T*

In the presence of nonzero temperature, the Dirac ring is smeared out over a radius of 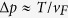. Furthermore, hole carrier from the 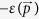 band also participate in current transport. The smearing of the Dirac ring results in qualitatively similar modifications to the response curve as increasing *μ* or *m*. However, the participation of the hole carriers undermines the destructive interference of 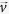 described in Fig. 2, and can destroy the characteristic non-monotonicity and thus negative differential resistance of the response curve at sufficiently high *T*. Nonetheless, we numerically find (Fig. 6 (Right)) that the ideal response curve retains its qualitative shape till *room* temperature (300K) for 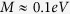, the inversion symmetry breaking scale in experimental realizations. This cutoff temperature is approximately 3000 *M* in units of *K*/*eV*.

#### Effect of scattering

Scattering is inevitable in real materials. For instance, a scattering time of 
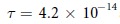
 s was reported for a Bi-based TI compound^36^. Here, we shall model the effect of scattering by a classical relaxation time *τ*, and show that its effects can be incorporated into our calculations by replacing the input driving field 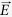 with a ‘renormalized’ *effective* driving field 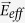.

The electronic states are distributed according to a time-dependent occupation function *g* (*p*(*t*), *t*) that obeys the Boltzmann equation


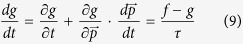


where 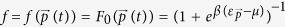 is the local equilibrium electron distribution, *F*_0_ being the Fermi-Dirac distribution which can deviate from *g* = *g*(*p*(*t*), *t*), the true, non-equilibrium distribution. Being the equilibrium distribution, the functional form of 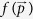 remains unchanged, and all fluctuations in time occurs only through the time-varying momentum of an electron 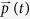. By contrast, *g* can deviate from this equilibrium through its explicit time dependence, although it will relax towards *f* with a characteristic time *τ*. We will assume spatial homogeneity throughout.

Eq. 9 has an explicit solution though elementary calculus:


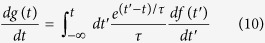


where the dependence on 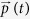 has been made implicit. Intuitively, 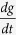 is a Laplace transform of 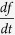, with contributions from earlier times exponentially suppressed due to scattering. As derived in Sect., Eq. 10 has an explicit solution:


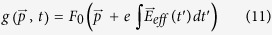


where


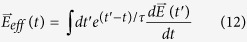


is the *effective* electric field that contains the effect of scattering. Essentially, 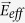 is contributed by past *increments* of the electric field 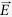 that are exponentially suppressed with a characteristic time *τ*. As *τ* increases, the increments have more time to constructively interfere. Physically, 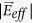 increases as *τ* increases because a longer ballistic motion contributes greater to the momentum shift of each particle. Of course, 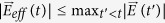. The manifest causality structure results from the time-reversal asymmetry of the system cf. Eq. 9.

For a simple sinusoidal driving field 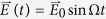, Eq. 12 (or Eq. 34) can be solved for the *exact* effective field


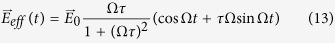


leading to the effective impulse


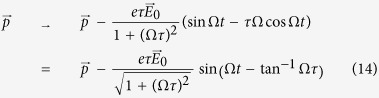


In other words, scattering leads to a renormalization of the amplitude by a factor 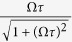 and a phase retardation corresponding to the duration 
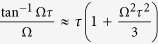
. The latter is trivial for a single frequency mode, but will lead to interference effects when there is a mixture of modes. That will further studied in Sect., in the limits of weak and strong scattering. Note that an expression very similar to Eq. 14 also appeared in ref. 19 in the context of radiation damping. But here *τ* has a more generic interpretation, encompassing all generic damping mechanisms.

The DC limit can be recovered by taking 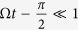, so that 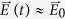. The effective impulse is equal to 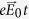 in the ballistic regime, but saturates at 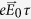 when *t* is increased to *τ* and effect of scattering is felt.

## Discussion

Through an approach based on the Boltzmann equation, we have analytically and numerically obtained the novel nonlinear response curve of a Dirac ring system representing Topological Insulator thin films. Due to the special topology of a ring-shaped region of filled states, the response curve is intrinisically non-monotonic, leading to a regime with negative differential resistance which heightens frequency upconversion of a periodic signal. This property remains robust in real, experimentally fabricated samples in the terahertz gap, with nonzero mass gap, chemical potential, temperature and impurity scattering rate.

One test of our results will be to reproduce the output signals in Fig. 4. To produce the *A*_0_ = 1 curve, for instance, we can input either an electrical or optical sinusoidal signal^37^ of amplitude 
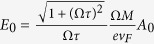
 on a sample of Bi_2_Se_3_ thin film thicker than 6 QLs. Experimentally obtained parameters 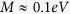, 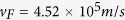 26, and 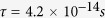 36, correspond to a realistic electric field of magnitude^37^ 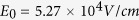 and frequency Ω = 100 *GHz*. The output current should closely agree with that in Fig. 4 at the temperature of liquid nitrogen *T* = 77 *K*, and deviate slightly from it at room temperature *T* = 300 *K* (cf. Fig. 6(Right)). All other output signal shapes in Fig. 4 may be reproduced by varying the input signal amplitude. Since the effective impulse from the field increases linearly with the scattering time *τ*, TIs with much larger *τ* will exhibit the aforesaid negative differential resistance and nonlinear response behavior at much weaker physical electric fields. This will be very attractive for low power applications, and is well on track to becoming a reality with the rapid development of TI material growth techniques as well as the discovery of novel TI materials.

## Methods

### The current response of a gapless Dirac ring

Here we derive the analytic expression for the response of an (ideal) gapless Dirac ring, for which the 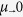 result in Eq. 6 is a special case.

Substituting the canonical velocity 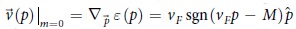 in Eq. 3, where 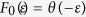 at temperature *T* = 0, we obtain


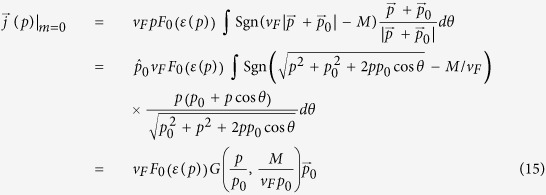


where *G*(*P, Q*), 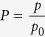, 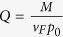 is a closed-form expression given by


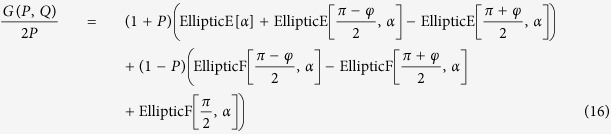


with 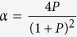, 
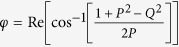
, 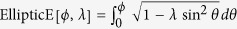 and 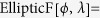 
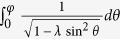
. The functional dependence 
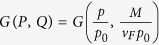
 implies that of the 3 parameters *p, p*_0_ and *M*/*v*_*F*_, only two can affect the result independently. For instance, the effect of letting 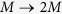 is equivalent to that of 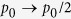, 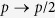 and 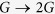.

We are now ready to calculate 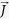 proper. It is just a sum over annuli of different radii *p*, each contributing a current 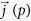. From Eq. 4, *μ* defines two Fermi surfaces with inner and outer Fermi momenta 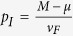 and 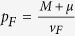. Considering the interesting case of small *μ* < *M*, the occupied momenta lie in a ring of inner and outer radii *p*_*I*_ and *p*_*F*_. In the limit of 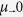, the occupied states form a very thin annulus and an illuminating closed-form expression exists for 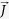:



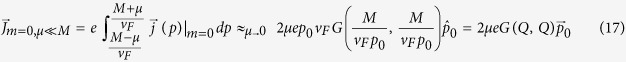

which is just Eq. 6. For larger *μ*, numerical integration yields the plots in Fig. 3 which has the following properties:

- The current is notably proportional to *v*_*F*_. This property is unique to the quasi-1D shape of the ring. While the velocity operator 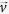 is proportional to *v*_*F*_, the area of the ring is independent of it. This is because its radius is proportional to it, while its thickness is inversely proportional to it.
- The response at any finite *μ* does not converge uniformly to Eq. 6: At small perturbations *p*_0_, the ring feels opposite velocity fields at both sides of the Fermi surface (FS), and there is a resultant linear regime. From Eq. 6, one can show that
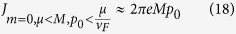
for 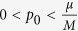 to a very high degree of accuracy, as shown in Fig. 3. Next comes a nonlinear regime where the response is negative approximately in the range *μ* < 0.6*v*_*F*_.
- At 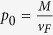 or *Q* = 1, we pass a special point where the ring untangles from the ring of Dirac nodes. Here,
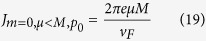
- For larger 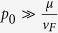 or *Q* ≫ 1, we have

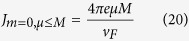


If *μ* ≥ *M*, the occupied momenta lie in a disk of radius *p*_*F*_ for the lower band, and of radius 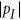 for the upper band. Note that all incompletely filled bands must be included. Of course, the model must be lattice regularized if we want to study huge *p*_*F*_ of the order of *π*.

It can also be shown that


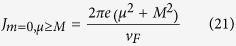


### Current response of a gapped Dirac ring

Here we derive some analytic results for the response due to small perturbations about a thin gapped Dirac ring, so as to understand how the ring structure affect the linear response of an otherwise massive system.

For sufficiently small *μ* − *m* and *p*_0_, the inequality 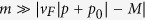 holds and the canonical velocity is approximately


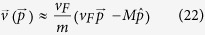


This is also what we have in the vicinity of the ring of minima of a system with Rashba splitting. Substituting it into Eq. 3 like before, we obtain


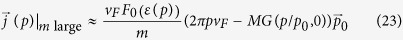


The first term is linear in 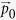, agreeing with that of usual materials with quadratic dispersion 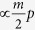


‡ where *m* is the effective mass; upon doing the *p* integral, we recover



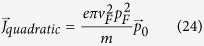

where *p*_*F*_ is the (outer) Fermi momentum. The second term is a Dirac-like contribution that modifies the response from the lowest-order quadratic approximation. Integrating both terms, we obtain



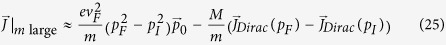

where 
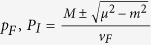
 and 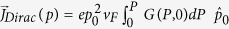 denotes the Dirac cone (*M* = 0) current in Eq. 3 and 16.

### The effective driving field due to scattering

Here we show that the non-equilibrium state distribution *g* is of the form


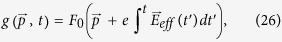


and derive 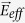 in terms of the original driving field 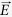. The Boltzmann Eq. 9 has an explicit solution Eq. 10:


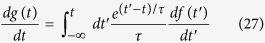


where 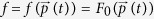. Care has to be taken in handling these PDEs: while *g* depends on 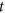 both explicitly and implicitly through 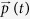, the dependence of *f* on *t* is only implicit through 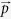 and 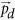, respectively before and after integrating out the damping effect. To motivate the solution to Eq. 10, we first solve it for a periodic driving field. For each fourier component Ω, we have 
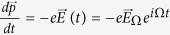
, so that


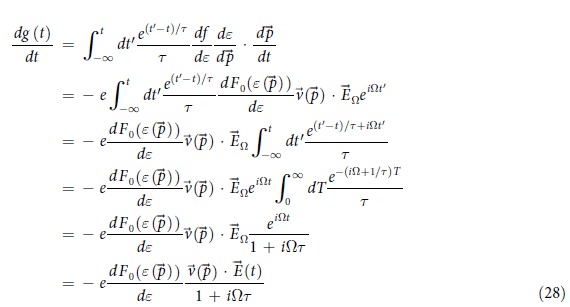


For a generic periodic driving field, Eq. 28 still holds, but with a sum over all fourier modes Ω. Hence the effect of the *τ* damping is the replacement of the fourier coefficients 
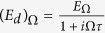
, which can also be guessed from elementary considerations.

As such, let’s define a *damped* momentum 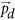 which responds to the damped electric field 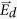 with fourier coefficients (*E*_*d*_)_Ω_. From Eq. 28, we obviously have


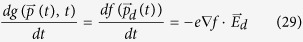


This integrates to


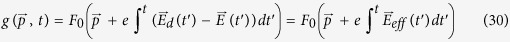


where


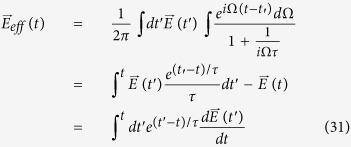


#### The diffusion limit of small *τ*

In the limit of strong scattering, only recent memories of 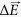 survive, and an expansion about *t*′ = *t* in Eq. 31 gives


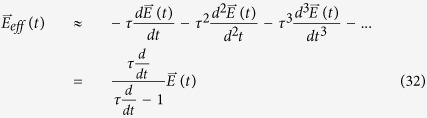


The above expansion is valid in the regime *τ* < Δ*t*, where 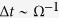 is the characteristic time scale at which 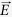 varies. To linear order, the current is thus


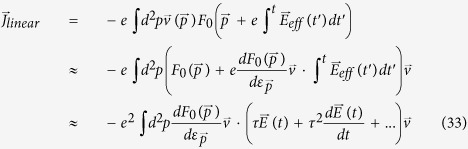


This linear approximation coincides exactly with the usual derivation of the Drude formula. If we allow for large perturbations, we will instead have


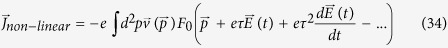


Clearly, the argument of *F*_0_ is the unperturbed crystal momentum 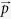 plus the impulse from 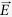 over an effective duration of *τ*. Note that it is *not* a Taylor expansion of 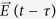; it is the series form of the exponentially suppressed field given in Eq. 31, with terms given by 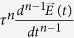.

#### The ballistic limit of large *τ*

First, we consider what happens when 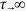, which is the limit studied in ref. 19. Since the system is driven periodically, 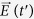 has equal positive and negative contributions, and


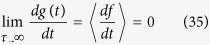


That 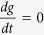 forces the *g* to have the functional form 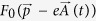, where explicit time dependence only enters through 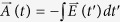. *F*_0_ is fixed to be the Fermi-Dirac distribution by considering the limit 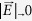. In a sense, this argument is a simple yet insightful semiclassical justification of the minimal substitution 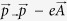 for a distribution: only through this substitution will *g* remain a constant as we follow a particle, as it should be in the absence of any other force.

Now, let us consider first-order scattering contributions 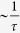. From the second line of Eq. 31, we have


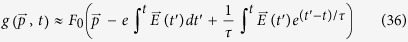


The 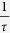 term keeps track of the effects of scattering. Note that is a Laplace transformation in 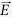 itself, and not of 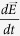 like in the diffusive limit.

## Additional Information

**How to cite this article**: Lee, C. H. *et al.* Negative differential resistance and characteristic nonlinear electromagnetic response of a Topological Insulator. *Sci. Rep.* **5**, 18008; doi: 10.1038/srep18008 (2015).
